# Pathogenic interplay between markedly elevated plasma lipoprotein(a) levels and prothrombotic mechanisms: a case report

**DOI:** 10.3389/fcvm.2026.1739634

**Published:** 2026-05-01

**Authors:** Marta Biolo, Angela Napolitano, Luca Spiezia, Cristiana Bulato, Serena Toffanin, Daniela Regazzo, Camilla Portinari, Alberto Zambon, Paolo Simioni

**Affiliations:** Department of Medicine, First Chair of Internal Medicine, Padova University Hospital - School of Medicine, Padova, Italy

**Keywords:** case report, extracellular vesicles, lipoprotein(a), platelet function assays, prothrombotic risk, thrombin generation, thromboelastometry

## Abstract

Lipoprotein(a) [Lp(a)] is a well-established genetic risk factor for atherosclerotic cardiovascular disease, though its role as a prothrombotic risk factor remains only partially understood. We report the case of a 64-year-old woman with markedly elevated Lp(a) levels (925 nmol/L, reference range < 105 nmol/L) and a history of recurrent major cardiovascular events, despite optimal lipid-lowering and antiplatelet therapies. We confirmed a hypercoagulable profile via comprehensive functional assessment of hemostasis: enhanced thrombin generation, reduced sensitivity to thrombomodulin, platelet hyperreactivity, and increased clot firmness at thromboelastometry — with residual platelet activity despite antiplatelet treatment. This case suggests a possible association between markedly elevated plasma Lp(a) levels and a hypercoagulable profile, which may enhance atherogenesis. Global coagulation and platelet function assays may help identify high-risk patients with elevated Lp(a) levels who may benefit from tailored antithrombotic strategies.

## Introduction

Lipoprotein(a) [Lp(a)] consists of a low-density lipoprotein (LDL)-like particle containing a single molecule of apolipoprotein B (apoB) 100, which is covalently bound to the glycoprotein apolipoprotein(a) [apo(a)] (1). It is well established that Lp(a) is a genetically determined risk factor for atherosclerotic cardiovascular disease (ASCVD) and calcific aortic valve disease. Mendelian randomization and epidemiological studies have demonstrated a causal association between elevated Lp(a) levels and ASCVD, including myocardial infarction, peripheral artery disease (PAD), ischemic stroke, and restenosis following percutaneous coronary intervention (2–5). The pathogenic role of Lp(a) is traditionally attributed to a dual functional mechanism: on the one hand, its structural similarity to low-density lipoproteins (LDL), which underlies its pro-atherosclerotic effects; on the other hand, its homology with plasminogen, which suggests a contribution to thrombosis by inhibiting fibrinolysis. Lipoprotein(a) is characterized by a high content of oxidized phospholipids, bioactive molecules capable of activating arterial endothelial cells, promoting the migration of monocytes and macrophages, and inducing the release of pro-inflammatory cytokines (6, 7). This cascade of events leads to persistent activation of inflammatory pathways, maintaining a chronic state of vascular inflammation and accelerating the progression of atherosclerosis (8). In addition to its inflammatory properties, Lp(a) appears to play an active role in thrombosis via multiple interconnected mechanisms that enhance coagulation activation, as well as thrombus formation and stabilization. The apolipoprotein(a) [apo(a)] component shares a structural homology with plasminogen but lacks its fibrinolytic activity, thereby interfering with endogenous fibrinolysis and contributing to the prothrombotic potential of Lp(a) (9). Furthermore, Lp(a) promotes coagulation by stimulating tissue factor (TF) expression and inhibiting the activity of its main regulator, tissue factor pathway inhibitor (TFPI), thus contributing to an increased thrombotic risk (9). Another emerging hypothesis focuses on the potential role of Lp(a) in modulating platelet function (10). It has been proposed that Lp(a) may directly influence platelet activity through interaction with specific platelet receptors, leading to the release of vasoactive substances and promoting platelet activation and aggregation. Recent evidence supports a possible association between circulating Lp(a) levels and platelet reactivity, which may contribute to the pathogenesis of cardiovascular disease (CVD) (11). Despite the growing interest, the underlying pathophysiological mechanisms through which lipoprotein (a) [Lp(a)] may exert its prothrombotic and pro-inflammatory effects have yet to be fully elucidated. In this report, we describe the case of a 64-year-old woman with markedly elevated plasma Lp(a) levels (925 nmol/L, reference range < 105 nmol/L) who experienced multiple major adverse cardiovascular events. The patient underwent an extensive diagnostic workup to assess the pathological interplay between Lp(a) and prothrombotic mechanisms, with a particular focus on primary hemostasis and platelet function.

## Case report

We report the case of a 64-year-old Caucasian female patient who was referred in June 2024 to the lipid clinic at Padova university hospital, for difficult-to-control hypercholesterolemia despite maximally tolerated lipid-lowering therapy. Her medical history was notable for type 2 diabetes mellitus and arterial hypertensions — both well controlled with pharmacological treatment — as well as a family history of hypercholesterolemia and late-onset cardiovascular disease.

In February 2024, the patient was admitted to the Emergency Department for acute coronary syndrome complicated by acute pulmonary edema. Coronary angiography revealed critical three-vessel coronary artery disease, and she subsequently underwent triple coronary artery bypass grafting. In addition, color doppler ultrasonography showed multivessel atherosclerotic disease, including asymptomatic PAD of the lower extremity, moderate bilateral renal artery stenosis, and mild carotid atheromatous changes without hemodynamically significant stenosis. Intracranial vascular imaging was not deemed necessary, as the patient did not present with neurological signs or symptoms suggestive of cerebrovascular insufficiency. At discharge, 12 days after hospitalization, she was prescribed dual antiplatelet therapy (DAPT) with aspirin and clopidogrel, in combination with lipid-lowering therapy with atorvastatin 20 mg once a day (OD) and ezetimibe 10 mg OD. The patient received a relatively low dose of atorvastatin due to a documented history of intolerance to high-intensity statin therapy resulting in statin-associated muscle symptoms. At the time of our outpatient evaluation, on this therapeutic regimen, her lipid profile was as follows: LDL-C 102 mg/dL, triglycerides 206 mg/dL, and high density lipoprotein cholesterol (HDL-C) 43 mg/dL. A further assessment of cardiovascular risk biomarkers showed apoB 1.01 g/L and markedly elevated Lp(a) levels at 925 nmol/L. High-sensitivity C-reactive protein (hs-CRP) levels were normal (1.81 mg/L; reference range 0.00–3.00 mg/L). Clinical assessment and routine laboratory investigations did not reveal any clinical or biochemical findings suggestive of an underlying malignancy or chronic infection.

In October 2024, the patient was readmitted after developing acute ischemia of the left lower extremity requiring urgent vascular surgery. The tibioperoneal trunk, posterior tibial artery, and peroneal artery were found to be occluded; therefore, a below-knee femoropopliteal bypass was performed, together with percutaneous transluminal angioplasty to treat a stenosis at the origin of the anterior tibial artery. At discharge, 17 days after hospitalization, dual antiplatelet therapy was maintained, and low-molecular-weight heparin (LMWH) (6000 IU twice daily) was added. Due to the patient's significantly high cardiovascular risk and progressive atherosclerotic disease with markedly elevated Lp(a), lipoprotein apheresis was proposed. In November 2024, the patient consented to undergo her first lipoprotein apheresis [i.e., direct adsorption of lipoproteins (DALI) 500]. The patient underwent blood sampling for hemostatic assessment in clinically stable conditions immediately before the first planned session of lipoprotein apheresis — she exhibited no acute cardiovascular or inflammatory events were present at sampling.

The procedure was terminated due to the onset of vasovagal syncope. The patient declined any additional lipoprotein apheresis thereafter. Consequently, lipid-lowering therapy was intensified by initiating a PCSK9 inhibitor with the goal of reducing LDL-C below 40 mg/dL, in accordance with the most recent European Society of Cardiology (ESC)/European Atherosclerosis Society (EAS) guidelines on the management of dyslipidemia available at that time (2019) (12). In addition, acetylsalicylic acid (ASA) dosage was increased from 100 mg to 160 mg OD, and anticoagulant therapy (LMWH 6000 IU SC twice daily) was maintained. Ten days after the unsuccessful lipoprotein apheresis, the patient experienced chest pain and died that night from a fatal myocardial infarction.

## Methods

After obtaining written informed consent and an overnight fast, blood samples were collected by venipuncture directly into five BD Vacutainer® citrate tubes [0.109 M (3.2%) sodium citrate, 9:1 ratio] using a 21-gauge needle and a light tourniquet, discarding the first milliliter. Platelet-poor plasma (PPP) was prepared within 1 h by double centrifugation (2,500  ×  g, 15 min, room temperature), aliquoted (0.5 mL), and stored at −80 °C until analysis. The techniques used to evaluate the effects of Lp(a) on primary and secondary hemostasis are described below.

The Platelet Function Analyzer-200 (PFA-200; Siemens Healthineers, Erlangen, Germany) measures platelet plug closure time (CT) under high shear conditions using collagen/epinephrine (COL/EPI) and collagen/ADP (COL/ADP) cartridges. Analysis was performed on citrated whole blood according to standard protocols, simulating the hemodynamic conditions of small injured vessels (13). The system uses single-use cartridges to assess different aspects of platelet reactivity and is useful for detecting platelet function disorders and monitoring antiplatelet therapy.

The Multiplate® analyzer (Roche Diagnostics GmbH, Mannheim, Germany) was used to assess Lp(a)-related changes in platelet aggregation. This whole-blood test measures electrical impedance between two electrodes, enabling rapid and sensitive monitoring of platelet function. Citrated blood (300 μL) was mixed 1:1 with saline at 37  °C and stimulated with arachidonic acid (ASPItest), ADP (ADPtest), or TRAP (TRAPtest) (14). Aggregation was recorded over 6 min and expressed as area under the curve (AUC, U; 1 U = 10 AU  ×  min), providing insights into Lp(a)-mediated thrombotic risk.

Light transmission aggregometry (LTA) was performed on platelet-rich plasma (PRP) following standard procedures (15, 16). PRP was obtained by centrifuging citrated blood at 150 × g for 10 min and adjusted to a standardized platelet count with autologous platelet-poor plasma. Aggregation assays were run on the CN-3000 Automated Analyzer (Siemens Healthineers) and expressed as aggregation curves (% light transmission). PRP without stimuli was 0% and PPP from the same patient was 100%. Agonists included collagen (2 μg/mL), ADP (10 μM), ristocetin (1.2 mg/mL), arachidonic acid (1 mM), and adrenaline (5 μM); values >70% were considered normal. For each test, 140 μL of PRP was used at 37 °C with magnetic stirring, and aggregation was recorded over 5 min.

Rotational thromboelastometry (ROTEM®, Werfen, Bedford, MA, USA) is a whole-blood viscoelastic test that evaluates coagulation from clot formation to lysis by measuring blood resistance under constant rotation. ROTEM® identifies coagulation changes associated with Lp(a), providing insight into hypercoagulability and platelet function. Analyses followed standard protocols (17, 18) and included INTEM (intrinsic pathway), EXTEM (extrinsic pathway), and FIBTEM (fibrinogen contribution). For INTEM and EXTEM, parameters were clotting time (CT, s; time to 2 mm), clot formation time (CFT, s; time from 2 to 20 mm), and maximum clot firmness (MCF, mm); FIBTEM considered MCF only. CT reflects initiation, CFT and alpha angle describe clot kinetics, and MCF indicates clot strength.

Thrombin generation (TG) assay was performed on whole blood within 4 h of collection, with or without thrombomodulin (TM, 20 nmol/L) (Synapse Research Institute, Maastricht, The Netherlands) as previously described (19, 20). Blood was mixed with the fluorogenic substrate Z-Gly-Gly-Arg-aminomethylcoumarin (Z-GGR-AMC; Bachem, Basel, Switzerland) and incubated 10 min at 37  °C. A mixture of tissue factor (TF; Innovin®, Siemens Healthineers), CaCl2, and MgCl2 was added (WB:substrate:TF = 3:1:2). Final well concentrations were 50% WB, 1 pmol/L TF, 6 mmol/L CaCl2, 3 mmol/L MgCl2, 416.7 μM ZGGR-AMC, ± TM. TM concentration was chosen to inhibit TG ∼50% in healthy controls. Samples were measured in triplicate and calibrated using *α*2-macroglobulin-thrombin complex (*α*2M-T, 300 nmol/L thrombin). Fluorescence was recorded at 37  °C every 6 s (Fluoroskan Ascent™, Thermo Labsystems) and analyzed for endogenous thrombin potential (ETP, nM·min), peak thrombin, lag time, time to peak, and velocity index.

### Extracellular vesicles (EVs) analysis

Platelet-poor plasma (PPP) was thawed at 37  °C for 5 min. Large EVs (L-EVs) were isolated by centrifugation at 14,000 × g for 30 min at 4  °C and immediately processed for immunolabeling after a single freeze-thaw cycle. Flow cytometry was performed on a CytoFLEX S cytometer (Beckman Coulter, USA) as previously reported (21). Size calibration used fluorescent Gigamix beads (0.1–0.9 μm), with side scatter from the 405 nm laser triggering detection (≥80 nm). L-EVs were labeled with calcein-AM (viability), Alexa Fluor 647 Annexin V (apoptosis), and antibodies against CD62E, CD62P, CD41, CD45, CD14, and tissue factor (TF), including isotype controls. Primary antibody incubation was 30 min at 37  °C, followed by 30 min at room temperature for secondary antibodies. Samples were resuspended in Annexin V buffer and analyzed within 1 h under constant acquisition settings. EVs were expressed as events/*μ*L and analyzed using CytExpert v2.3. True EVs were defined as calcein-AM positive plus one specific marker; triple-positive EVs (calcein+/CD62E+/TF+) were also assessed. This method characterizes EVs and their procoagulant potential, helping evaluate Lp(a)-related thrombogenicity for cardiovascular risk assessment.

## Laboratory results

### Complete blood count

Mild normocytic, normochromic anemia (hemoglobin 113 g/L, reference range 120–153 g/L), with a normal platelet count (415 x10⁹/L, reference range 150–450 x10⁹/L).

### Coagulation profile

Hemostatic assessment showed a prothrombin time (PT) ratio of 1.09 (reference range 0.8-1.2), an activated partial thromboplastin time (aPTT) ratio of 1.18 (reference range 0.8-1.2), and a fibrinogen level of 3.98 g/L (reference range 2.0-4.0 g/L). Antithrombin activity was within the reference range (118%, reference range 80%-120%). Plasma homocysteine levels were normal (8.2 µmol/L; reference range 5–12 µmol/L), and the coagulation profile did not suggest a clinically significant inherited or acquired thrombophilia. In particular, protein C levels (150%) and coagulometry parameters (130%) were elevated, findings generally considered non-pathological and not associated with increased thrombotic risk. Protein S demonstrated a mildly reduced free fraction (66%; reference range 70-160%) with normal functional activity (99%), a pattern not consistent with severe deficiency. Tissue factor pathway inhibitor (TFPI) levels were normal (94 ng/mL; reference range 70–100).

### Platelet function analyzer-200 (PFA-200)

The COL/EPI test demonstrated a closure time within the reference range (133 s; reference range 82-150 s), whereas the COL/ADP test resulted in immediate closure.

### Multiplate® analyzer

The ASPItest value was slightly below the lower limit of the reference range, 47 U (reference range: 51-112 U), suggesting partial inhibition of the arachidonic acid pathway by ASA, whereas the ADPtest and TRAPtest values remained within the reference range, indicating preserved platelet function in pathways not directly targeted by ASA's mechanism of action (Table 1).

**Table 1:** ROTEM® thromboelastometry and multiplate aggregometry in a 64-year-old female patient with markedly elevated Lp(a) levels.

Whole blood assays	Patient with high Lp(a)	Reference range
ROTEM thromboelastometry		
Clotting time (CT), sec		
INTEM	193	161-204
EXTEM	65	50-80
Clot formation time (CFT), sec		
INTEM	48	62-130
EXTEM	43	46-149
Maximum clot firmness (MCF), mm		
INTEM	76	51-69
EXTEM	76	51-69
FIBTEM	26	55-72
Multiplate aggregometry		
ADP-test	104	48-119
ASPI-test	51	50-112
TRAP-test	121	86-159

### Light transmission aggregometry (LTA)

Results demonstrated a dose-dependent response to ADP (68.8% at 2 µM and 81.1% at 10 µM), a moderately reduced aggregation response to collagen (50.2%), and a notably pronounced reduction in response to epinephrine (36.8%). Aggregation induced by arachidonic acid was markedly diminished (18.2%), consistent with antiplatelet therapy with ASA. Aggregation induced by ristocetin was preserved (82.8%), indicating intact platelet–von Willebrand factor-endothelium.

Rotational thromboelastometry (ROTEM®) demonstrated a markedly hypercoagulable profile, characterized by a shortened propagation phase of coagulation and increased clot firmness (Table 1). Specifically, a reduced clot formation time (CFT) was observed in both EXTEM (43 s; reference range 46-149 s) and INTEM (48 s; reference range 62-130 s). Maximum clot firmness (MCF) was elevated in EXTEM (76 mm; reference range 55-72 mm), INTEM (76 mm; reference range 51-69 mm), and FIBTEM (28 mm; reference range 6-21 mm), indicating increased clot strength.

### Thrombin generation (TG)

**Figure 1:**
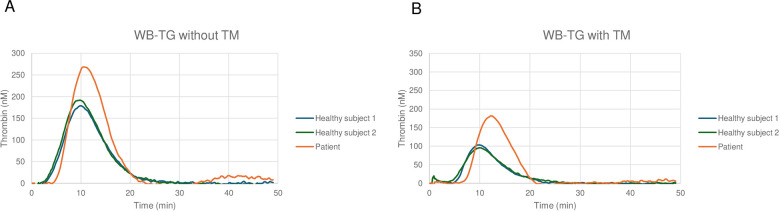
Whole blood thrombin generation (WB-TG) without **(A)** and with **(B)** thrombomodulin (TM) in a 64-year-old female patient with markedly elevated Lp(a) levels.

The patient's measurements were compared to those of an historical group of 20 healthy subjects described previously (20) (Table 2 and Figure 1). The patient's endogenous thrombin potential (ETP) was 2,116.5 nM·min, a value above the 75th percentile of the healthy cohort (1,804.2 nM·min), indicating an increased thrombin-generating capacity. Similarly, the ETP in the presence of thrombomodulin (ETP + TM) was elevated (1,374.0 nM·min), above the 75th percentile of controls (1,137.5 nM·min). The percentage inhibition of ETP, which reflects the effectiveness of thrombomodulin-mediated anticoagulant control, was reduced in the patient (35.1%) compared to the median value among the healthy cohort (46.6%), suggesting a deficiency in endogenous anticoagulant regulation. The patient had elevated values of thrombin peak generation (260.5 nM), above the 75th percentile of controls (228.9 nM), whereas the percentage inhibition of the peak by thrombomodulin was markedly lower (29.1 vs. 41.0%), highlighting a hypercoagulable state. Latency time (lag time) and time to peak were longer in the patient (5.1 and 10.9 min, respectively) compared to the median value among healthy controls (3.8 and 9.7 min, respectively), indicating an initial delay in the coagulation process followed by a rapid peak generation phase. Finally, the velocity index of thrombin generation (vel index) was higher in the patient (45.0 nM/min) compared to controls (36.4 nM/min), confirming a faster thrombin growth phase.

**Table 2:** Whole blood thrombin generation (WB-TG): endogenous thrombin potential with and without thrombomodulin in a 64-year-old female patient with markedly elevated Lp(a) levels vs. healthy individuals.

WB thrombin generation	Patient with high Lp(a)	Healthy subjects (*n* = 20)
ETP (nM*min)	2,116.5	1,614.5 (1,373.6–1,804.2)
ETP + TM (nM*min)	1,374.0	895.2 (754.9–1,137.5)
Peak (nM)	260.5	197.4 (175.6–228.9)
Peak + TM (nM)	184.5	117.3 (109.6–132.7)
Lag time (min)	5.1	3.8 (3.2–4.3)
Lag time + TM (min)	7.1	5.9 (5.2–6.5)
Time to peak (min)	10.9	9.7 (8.3–10.0)
Time to peak + TM (min)	12.0	10.2 (9.5–11.5)
Vel index (nM/min)	45.0	36.4 (29.3–39.7)
Vel index + TM (nM/min)	37.7	30.6 (24.5–37.6)
ETP inhibition (%)	35.1	46.64 (33.42–52.49)
Peak inhibition (%)	29.2	41.02 (30.64–44.13)

Median values are reported with 25th and 75th percentile values in parenthesis. ETP, endogenous thrombin potential; HS, healthy subjects; TM, thrombomodulin.

### Extracellular vesicles (EVs) analysis

Extracellular Vesicles (EVs) analysis. The patient's measurements were compared to those of an historical group of 20 healthy subjects described previously (20) (Table 3). The analysis of microvesicles revealed a general trend towards the reference range across most EVs populations examined. In particular, platelet-derived microvesicles were within the reference range overall, except for a moderate decrease in Calcein + CD41 + EVs, suggesting a possible reduction in platelet-derived EVs populations, and a marked decrease in Calcein + AnnexinV + CD41 + CD62P + EVs, indicating low levels of platelet activation and apoptosis — both processes that are crucial for the secretion of this subpopulation. The patient had significantly low levels of Calcein + AnnexinV + CD41 + EVs, potentially indicating significant quantitative or functional alterations in early apoptotic platelet-derived EVs. We also observed a reduction in angiogenesis-related EVs, in particular CalceinRED + CD105 +, AnnexinV + CD105 + and CalceinRED + AnnexinV + . Endothelial cell-derived EVs also showed a widespread reduction compared to reference values, affecting both viable (Calcein +) and apoptotic (AnnexinV +) subpopulations, with or without TF expression. Only the Calcein + Annexin + CD62E + TF + EVs levels remained within the reference range, albeit at the lower end. Lastly, levels of both viable and apoptotic inflammatory EVs — i.e., CD45 + and CD14 + subpopulations — were reduced compared to reference means.

**Table 3:** Large EV subgroups levels in a 64-year-old female patient with markedly elevated Lp(a) levels, acquired using the CytoFLEX flow cytometer (beckman coulter).

Large EV subgroups	Patient with high Lp(a)	Healthy subjects (*n* = 20)
Platelet panel		
Calcein + CD41 + CD62P+	153.26	152.73 ± 99.84
Calcein + CD62P+	373.96	508.69 ± 287.02
Calcein + CD41+	555.84	1,082.32 ± 456.60
Annexin + CD62P+	263.61	394.18 ± 209.01
AnnexinV + CD41+	719.32	1,020.61 ± 454.44
Calcein + AnnexinV + CD41 + CD62P+	153.60	1,417.64 ± 522.09
Calcein + AnnexinV + CD41+	89.91	45,715.17 ± 3,851.56
Angiogenesis panel		
CalceinRED + CD105+	2.00	27.00 ± 17.28
AnnexinV + CD105+	4.05	17.05 ± 6.36
CalceinRED + AnnexinV+	273.60	394.39 ± 179.38
Endothelial panel		
Calcein + CD62E+	115.79	541.82 ± 27.28
Annexin + CD62E+	14.22	50.17 ± 26.62
Calcein + TF+	16.90	42.47 ± 25.57
AnnexinV + TF+	339.24	813.95 ± 423.94
Calcein + Annexin + CD62E + TF+	6.09	14.99 ± 11.34
Inflammation panel		
Calcein + CD14+	962.64	1,481.46 ± 789.76
Calcein + CD45+	851.99	1,254.52 ± 695.62
Calcein + AnnexinV+	303.18	614.14 ± 263.13
Annexin + CD45+	225.72	691.16 ± 300.44
Annexin + CD14+	298.75	686.27 ± 281.65
Calcein + AnnexinV + CD45 + CD14+	216.87	376.27 ± 151.27

Data (events/μL) are expressed as mean values vs. healthy subjects (mean ± standard deviation). EV, extracellular vesicle; TF, tissue factor; CD41, platelet marker; P-selectin/E-selectin, endothelial or platelet activation markers; CD45, leukocyte marker; CD14, monocyte marker; Calcein, viability dye.

## Discussion

The coagulative profile of our patient revealed a clear prothrombotic tendency and a suboptimal response to antiplatelet therapy. This suggests a possible direct pathogenic association between the markedly elevated plasma Lp(a) levels and both primary and secondary hemostasis.

According to the PFA-200 results, the patient had a normal closure time with COL/EPI, whereas the COL/ADP test resulted in immediate closure — which may have stemmed from very high Lp(a) levels resulting in altered blood rheology. High concentration of Lp(a) particles can increase plasma viscosity and affect red blood cell deformability, thus impairing microvascular flow. Combined with platelet hyperreactivity, this could lead to slowed or obstructed flow during the assay. In parallel, platelet function tests performed with impedance and LTA aggregometry apparatus (e.g., Multiplate® analyzer and CN3000, respectively) showed a partial response to ASA therapy, with an ASPItest value just below the reference range and significant residual aggregation with ADPtest and TRAPtest. Notably, we observed an attenuated response to epinephrine and arachidonic acid, consistent with a residual platelet activity despite antiplatelet therapy.

Interestingly, ROTEM® and thrombin generation assays, which were both conducted on whole blood, demonstrated a hypercoagulable pattern. In particular, ROTEM® showed reduced clot formation times (shortened CFT in INTEM and EXTEM) and increased clot firmness (elevated MCF in INTEM, EXTEM and FIBTEM). This pattern suggests enhanced clot strength mediated by both cellular components and fibrinogen. The results of whole blood thrombin generation further support the presence of a hypercoagulable state. In particular, the patient exhibited significantly higher ETP and thrombin peak values compared to healthy controls, along with reduced inhibition by thrombomodulin, suggesting a reduced efficacy of the thrombomodulin-mediated anticoagulant system. These findings support a prothrombotic state, consistent with the Lp(a)-mediated activation of the tissue factor pathway as described in the literature (22).

The evaluation of plasma EV levels showed a general trend towards the reference range. However, these findings should be interpreted with caution, as EV release can be affected by statin therapy, potentially masking underlying cellular activation (23). In particular, the lower plasma levels of platelet- and endothelium-derived EVs are consistent with reduced expression of cell surface activation markers observed in patients receiving lipid-lowering therapy (24). Similarly, the decreased levels of monocyte- and leukocyte-derived EVs, along with normal hs-CRP values, align with recent evidence suggesting that elevated Lp(a) may contribute to myocardial infarction independently of baseline inflammation (25). Overall, these observations are to be considered descriptive and exploratory in nature, and while they may hint at a predominantly Lp(a)-mediated thrombotic pathway rather than an inflammation-driven mechanism, definitive mechanistic conclusions cannot be drawn from this dataset. Our data indicates that elevated levels of Lp(a) not only drive atherogenesis but may interfere with the thrombotic homeostasis. The observed changes in platelet function tests, ROTEM® and thrombin generation assays may serve as early functional biomarkers of thrombotic risk in Lp(a)-mediated atherosclerosis. These diagnostic tools could prove invaluable in identifying patients who may benefit from more aggressive or tailored antithrombotic interventions.

We would remiss not to mention some of the limitations of our study. This report is inherently limited by its single-patient, case-based nature, which does not allow for the generalizability of the findings and precludes the establishment of any causal relationship. Nevertheless, the peculiarity of this case lies in the exploration of the potential pathogenic interplay between markedly elevated plasma Lp(a) levels and prothrombotic mechanisms. Although blood samples were collected when the patient was deemed clinically stable, we cannot definitively exclude that the proximity to recent acute vascular events may have influenced some coagulation parameters, particularly thrombin generation assays. In addition, normal hs-CRP levels and the lack of assessment of other systemic inflammatory biomarkers further preclude any definitive conclusions regarding a systemic pro-inflammatory state. Finally, we acknowledge that the lack of a detergent-based lysis control (e.g., Triton X-100) is a limitation of this study; however, stringent gating, size calibration, and specific surface markers were applied to minimize potential artifacts.

By providing an in-depth assessment of coagulation and platelet function, it offers hypothesis-generating insights that may guide future research on this topic. Another missing datapoint is the assessment of the patient's platelet function before initiating antiplatelet therapy, which may have helped ascertain a potential predisposition to platelet hyperreactivity or hyperaggregability, which may have contributed to the prothrombotic phenotype associated with elevated Lp(a). Finally, due to the patient's rapid clinical deterioration, we were unable to assess whether increasing the ASA dose from 100 mg to 160 mg orally once daily would have improved platelet aggregation parameters. Genetic testing for clopidogrel resistance was not performed. Nevertheless, this assessment may be relevant in future studies comprising patients with recurrent thrombotic events or progressive atherosclerotic disease despite adequate antithrombotic treatment.

In conclusion, this case highlights a complex prothrombotic phenotype observed in a patient with markedly elevated Lp(a) levels, characterized by: i) insufficient platelet inhibition despite ASA therapy; ii) enhanced whole blood thrombin generation; iii) impaired endogenous anticoagulant capability. However, due to the limited data currently available in the literature, the precise mechanisms underlying this hemostatic imbalance remain incompletely understood and warrant further investigation to inform the development of new diagnostic strategies and patient-tailored therapies in this high-risk population. Although this is only a case report and we thus acknowledge that no general conclusions can be drawn, we believe that even the fatal clinical outcome may contribute to highlighting the potential clinical relevance of this peculiar hypercoagulable profile. These observations are hypothesis-generating and do not allow for any inference of causality though they may help inform future mechanistic and clinical studies.

## Data Availability

The original contributions presented in the study are included in the article, further inquiries can be directed to the corresponding author.
